# Online Cognitive Stimulation Therapy for Dementia in Brazil and India: Acceptability, Feasibility, and Lessons for Implementation

**DOI:** 10.2196/55557

**Published:** 2024-06-11

**Authors:** Emily Fisher, Shreenila Venkatesan, Pedro Benevides, Elodie Bertrand, Paula Schimidt Brum, Céline El Baou, Cleusa P Ferri, Jane Fossey, Maria Jelen, Jerson Laks, Lisa Liu, Daniel C Mograbi, Nirupama Natarajan, Renata Naylor, Despina Pantouli, Vaishnavi Ramanujam, Thara Rangaswamy, Raquel L Santos de Carvalho, Charlotte Stoner, Sridhar Vaitheswaran, Aimee Spector

**Affiliations:** 1 University College London London United Kingdom; 2 Dementia Care in Schizophrenia Research Foundation Chennai India; 3 Pontifical Catholic University of Rio de Janeiro Rio de Janeiro Brazil; 4 Université Paris Cité Paris France; 5 Universidade Federal de Sao Paulo Sao Paulo Brazil; 6 University of Exeter Exeter United Kingdom; 7 Federal University of Rio de Janeiro Rio de Janeiro Brazil; 8 University of Pittsburgh Pittsburgh, PA United States; 9 Universidade do Grande Rio Rio de Janeiro Brazil; 10 University of Greenwich London United Kingdom

**Keywords:** psychosocial, intervention, technology, COVID-19, LMIC, low and middle income countries

## Abstract

**Background:**

Cognitive stimulation therapy (CST) is an evidence-based, group psychosocial intervention for people with dementia, and it has a positive impact on cognition and quality of life. CST has been culturally adapted for use globally. It was developed as a face-to-face intervention but has recently been adapted for online delivery.

**Objective:**

In this study, we aimed to explore the feasibility and acceptability of online or virtual CST (vCST) delivery in India and Brazil, emphasizing barriers and facilitators to implementation.

**Methods:**

A single-group, multisite, mixed methods, feasibility study was conducted, with nested qualitative interviews. Primary feasibility outcomes were recruitment rate, attendance, attrition, acceptability, and outcome measure completion. Exploratory pre- and postintervention measures, including cognition and quality of life, were assessed. Qualitative interviews were conducted with people with dementia, family caregivers, and group and organizational leaders following intervention delivery, and the data were analyzed using the Consolidated Framework for Implementation Research.

**Results:**

A total of 17 vCST group sessions with 59 participants were conducted for 7 weeks, with 53% (31/59) of participants attending all 14 sessions. Attrition rate was 7% (4/59), and outcome measure completion rate at follow-up was 68% (40/59). Interviews took place with 36 stakeholders. vCST was acceptable to participants and group leaders and enabled vital access to services during pandemic restrictions. While online services broadened geographic access, challenges emerged concerning inadequate computer literacy, poor technology access, and establishing interpersonal connections online. Exploratory, uncontrolled analyses indicated positive trends in quality of life but negative trends in cognition and activities of daily living, but these results were not statistically significant.

**Conclusions:**

vCST demonstrated feasibility and acceptability, serving as a crucial resource during the pandemic but raised challenges related to technology access, computer literacy, and long-term implementation. The study highlights the potential of vCST while emphasizing ongoing development and solutions to address implementation challenges.

## Introduction

### Background

Dementia affects more than 57.4 million people worldwide [1]. People with dementia in low- and middle-income countries (LMICs) make up 60% of all global cases [2]; however, high-income countries account for around 74% of global expenditure on dementia [3]. There are an estimated 1.8 million people >60 years of age with dementia in Brazil [4] and 8.8 million in India, representing 5.8% and 7.4% of people >60 years of age, respectively [4-6]. While demographic and socioeconomic factors differ between Brazil and India, both countries experience low diagnosis rates, limited access to specialist treatment and care, high levels of stigma, and a lack of dementia awareness [7-9].

Worldwide, pharmacological treatments for dementia remain limited, so nonpharmacological interventions are needed to address cognitive and behavioral symptoms of dementia and improve quality of life for people with dementia and their families [10]. Many interventions are developed, but only a few are delivered at large scale and have been adopted in routine practice [11].

Cognitive stimulation therapy (CST) is a brief, manualized group program which has been found to improve cognition and quality of life in people with mild to moderate dementia [12]. It comprises themed activities that stimulate and engage participants in a social group environment led by a trained facilitator through tasks such as physical activity, word association, and discussion of current affairs [13]. Despite evidence for its effectiveness [12], cost-effectiveness [14], and its successful cultural adaptation internationally in more than 35 countries [15], CST is yet to be implemented in routine practice outside of the United Kingdom [16,17]. The 2022 World Alzheimer’s Report recommended further research and implementation of CST globally [18].

This study was conducted as part of the CST-International research program, which explored the implementation of CST in 3 LMICs [19]: Brazil, an upper middle-income country; (2) India, a lower middle-income country; and (3) Tanzania, a low-income country [20]. In each country, research teams had previously translated and culturally adapted CST and carried out feasibility studies of face-to-face CST [16,17].

CST was developed as a face-to-face intervention. However, during the CST-International study, access to face-to-face health care services was rapidly restricted due to the COVID-19 pandemic [21]. CST started to be delivered virtually in international settings including the United Kingdom and New Zealand [22,23], but a standardized protocol was not available. An international collaboration resulted in a framework for global delivery, which was field-tested in Brazil and India, alongside the United Kingdom, Hong Kong, and Ireland [24].

### Objectives

This study aimed to build upon the initial development and field-testing of the online or virtual CST (vCST) protocol in Brazil and India, with a focus on feasibility, acceptability, and implementation. Therefore, the aims of this study were to (1) explore the acceptability of vCST to people with dementia, family caregivers, CST facilitators, and service managers in Brazil and India; (2) test the feasibility of online recruitment, delivery, and assessment for CST in Brazil and India; and (3) explore factors affecting the implementation of vCST.

## Methods

### Design

This study was a single-group, multisite, mixed methods, feasibility study, with pre- and postintervention outcome measures followed by semistructured interviews with people with dementia, caregivers, and intervention group leaders.

### Methodological Framework

This study is guided by the Consolidated Framework for Implementation Research (CFIR) [25]. The CFIR is a determinant framework, which incorporates domains that are hypothesized or found to influence implementation outcomes and has been applied in LMIC settings [26]. The five domains relate to (1) intervention characteristics (eg, evidence strength and quality as viewed by stakeholders and its core and adaptable components); (2) outer setting (eg, local attitudes to the condition of interest as well as external partnerships and financing); (3) inner setting (eg, available organizational resources and staffing); (4) characteristics of individuals involved in implementation, their need for the intervention and their capability, availability, and motivation to be involved (based on the capability, opportunity, and motivation behavior model) [27]; and (5) process of implementation (eg, assessing needs of intervention recipients, planning, and tailoring strategies). Use of the CFIR as a deductive qualitative framework enables comparisons of barriers and facilitators in other settings and for other interventions.

### Participants

People with mild to moderate dementia, supported by their family caregivers, took part in vCST group sessions. For the qualitative component, people with dementia, caregivers, group leaders, and organizational decision makers were invited to participate in qualitative interviews following the completion of the vCST groups.

### Setting

In Brazil, the study site was a psychology department at a university in Rio de Janeiro. In India, the study site was a mental health nongovernmental organization (NGO) in Chennai, offering outpatient, inpatient, and day center services for people with dementia. Both sites had previously been involved in cultural and virtual adaptation of CST [16,17,24] and therefore already had access to face-to-face CST manuals, vCST guidance, and CST trainers and facilitators.

### Recruitment

In Brazil, recruitment took place through partnerships with memory clinics and NGOs, advertisements on social media and local media, and snowball sampling. In India, people with dementia were recruited from the patient caseload at the NGO, with additional promotion through caregiver support groups and mobile-messaging groups.

The inclusion criteria for people with dementia at both sites were as follows: they must (1) meet the ICD-10 criteria for dementia as assessed by a trained clinician [28], (2) be rated as having mild to moderate dementia on the Clinical Dementia Rating Scale [29], (3) have sufficient hearing and vision to follow conversation and comment on visual material, and (4) have the ability to participate in a online group for 1 hour.

### Intervention Procedure

People with dementia were allocated to a vCST group. The vCST intervention was delivered according to the culturally adapted CST Brazil and India manuals [16,17]*,* which had been further adapted according to the recently developed protocol for online delivery of CST [24]. Groups took place twice weekly over 7 weeks via Zoom videoconferencing software (Zoom Video Communications) between February 2021 and September 2022. Each group consisted of 3 to 5 participants. In Brazil, participants needed to use their own devices, whereas in India, devices were loaned from the NGO if needed. In Brazil, the language of instruction was Brazilian Portuguese, and in India, it was Tamil or English. Group facilitators were trained by site leaders and worked in pairs to deliver the intervention. In Brazil, the group leaders were 2 psychologists, a gerontologist, and 8 trainee psychologists. In India, the group leaders were 3 psychologists and a nursing assistant.

### Feasibility Outcomes

The following prespecified main outcomes relating to intervention acceptability were adapted from the study by Proctor et al [30]:

- Recruitment rate: the recruitment target of 50 participants in Brazil and 15 in Chennai was intended to be achieved by September 2022. This target was calculated pragmatically based on available time and resources in each site and was deemed suitable to run enough vCST groups to explore feasibility, acceptability, and implementation issues.
- Attendance: overall attendance rate of >60%, based on the international team’s judgment and experience of running CST groups and supporting people with dementia in each setting.
- Attrition: retention rate of at least 75% of participants to the follow-up, again based on the team’s previous experience and judgment.
- Acceptability of intervention: this will be assessed through semistructured interviews (see the
  Semistructured Interviews
  section).
- Outcome measure completion: the inclusion of cognition, quality of life, activities of daily living, and caregiver burden measures are in line with those used in previous trials and studies of CST [12]. The main goal of the outcomes was to assess the feasibility and acceptability of collecting these data.

Outcome measures were translated, back-translated, and finalized by bilingual committee review. The following pre- and postintervention measures were completed by people with dementia:

- The Alzheimer’s Disease Assessment Scale-Cognitive Subscale, an internationally used 21-item measure of cognitive function [31]
- The World Health Organization Quality of Life Brief Version, a 26-item quality-of-life measure addressing 4 domains: physical health, psychological health, social relationships, and environment, which was developed for use in LMICs [32]
- Alzheimer’s Disease Cooperative Study-Activities of Daily Living Scale, a 23-item scale that addresses basic and instrumental activities of daily living and has been culturally adapted for use in Brazil [33,34]. This scale was used in Brazil only
- Scale for the Instrumental Activities of Daily Living in the Elderly, an 11-item scale developed in South India that addresses cognitive and physical disability [35]. This scale was used in India only

Family caregivers completed the following:

- The Zarit Burden Interview (ZBI), a widely used 22-item self-report measure of strain and stress [36].
- Dementia Caregiver Experience Scale, a 17-item measure to assess stress and strain (personal communication by Vaitheswaran, 2023), was included due to previous issues with the cross-cultural validity of the ZBI, which has been found to underestimate burden in LMIC settings [37].

### Semistructured Interviews

To gather in-depth information about intervention acceptability, feasibility, and experiences of implementation, semistructured interviews were conducted with stakeholders. People with dementia and caregivers from the first 2 vCST groups in Brazil (12 dyads) and all vCST groups in India (15 dyads) were invited to take part in dyadic interviews. A convenience sample of group leaders from Brazil and India (n=7) and organizational decision makers (n=2, India only) was invited to take part in one-to-one interviews.

Interviews with people with dementia and caregivers were conducted in the language of vCST group instruction (English, Tamil, or Brazilian Portuguese) and were led by a researcher from the respective institution who had not facilitated the group to reduce response bias. Interviews with group leaders and organizational decision makers were conducted in English. To reduce response bias, these were led by UK-based researchers who were not members of the immediate project team. All interviews took place over videoconferencing software.

On the basis of constructs from the CFIR, the interview guide was developed by researchers and clinical psychologists, with questions relating to the participants’ experience of taking part in the vCST group, experience of using a online platform, and barriers and facilitators to involvement.

### Analysis

#### Quantitative

Primary analysis was descriptive and assessed recruitment, retention, and outcome measure completion. The pre- and postintervention outcome measures, that is, means and mean differences (calculated through paired 2-tailed *t* tests), were reported descriptively. Effect sizes were calculated using Cohen *d*. Statistical analysis was performed using SPSS (version 29; IBM Corp). All data were pseudoanonymized with a unique participant identification number.

#### Qualitative

Transcripts in Brazilian Portuguese or Tamil were translated before analysis. First, the researchers read the transcripts for data familiarization. We used a framework analysis approach to code the transcripts using inductive thematic analysis [38] and mapped inductive themes onto the CFIR. This enabled us to capture themes that were not covered by the CFIR. Transcripts were coded independently by 2 researchers using NVivo software (Lumivero), who met regularly to ensure that they were approaching the data in a similar way and then agreed on a final coding framework. Any discrepancies were examined and resolved through discussion. An exception is the interview transcripts of caregivers in India, as it was coded by an individual reviewer using Atlas software (Observational Health Data Sciences and Informatics) and discussed with a second researcher.

### Ethical Considerations

Ethics approval was granted by the relevant body in each country. In Brazil, an ethics amendment was granted by the Federal University of Rio de Janeiro Institute of Psychiatry research ethics committee (ref: 57019616.5.1001.5263). In India, approval was granted by the institutional ethics committee at Schizophrenia Research Foundation; SCARF: Chennai (SRF-CR/17/0CT-2020). Informed consent was received from people with dementia and their caregivers upon recruitment. Participants did not receive compensation.

Interviews were recorded with consent and transcribed. All transcripts were pseudoanonymized with a unique participant ID number, and any identifying information was removed during transcription.

## Results

### Feasibility Outcomes

#### Recruitment Rate

A total of 59 participants were recruited to vCST groups, which was 91% of the target figure of 65 participants. Participant demographics are outlined in Table 1. In Brazil, 12 group sessions took place with a total of 44 participants between April 2021 and November 2022. In India, 5 group sessions were conducted with 15 participants between February 2021 and February 2022. This represents an average of 3.6 participants per group across both sites.

**Table 1:** Participant demographics.

				Brazil (n=44)		India (n=15)		Total (N=59)
**Person with dementia**								
	**Region, n (%)**							
		Southeast Brazil	41 (93)		—^a^		41 (69)	
		South Brazil	2 (5)		—		2 (3)	
		Northeast Brazil	1 (2)		—		1 (2)	
		Chennai (India)	—		15 (100)		15 (25)	
	Age (y), mean (range)		77.1 (61-93)		77.3 (65-93)		77.2 (61-93)	
	**Sex, n (%)**							
		Male	10 (23)		10 (67)		20 (34)	
		Female	34 (77)		5 (33)		39 (66)	
	**Ethnicity, n (%)**							
		South Asian	—		15 (100)		15 (25)	
		Black	3 (7)		—		3 (5)	
		Mixed	6 (14)		—		6 (10)	
		White	35 (80)		—		35 (59)	
	Education (y), mean (range)		11.5 (4-20)		13.0 (10-17)		11.9 (4-20)	
	**Type of dementia, n (%)**							
		Alzheimer disease	22 (50)		11 (73)		33 (60)	
		Vascular dementia	4 (9)		3 (2.0)		7 (12)	
		Mixed dementia (Alzheimer disease and vascular dementia)	1 (2)		1 (7)		2 (3)	
		Parkinson-related dementia	0 (0)		0 (0)		0 (0)	
		Dementia with Lewy bodies	1 (2)		0 (0)		1 (2)	
		Variant unknown	16 (36)		0 (0)		16 (27)	
**Caregiver**								
	**Sex, n (%)**							
		Male	7 (16)		3 (20)		10 (17)	
		Female	37 (84)		12 (80.0)		49 (83)	
	Age (y), mean (range)^b^		52.5 (32-71)		53.2 (29-72)		52.7 (29-72)	
	**Relationship to person with dementia, n (%)^c^**							
		Spouse	8 (19)		4 (27)		12 (20)	
		Daughter or son	32 (74)		11 (73)		43 (73)	
		Daughter-in-law or son-in-law	1 (2)		0 (0)		1 (2)	
		Other relative	2 (5)		0 (0)		2 (3)	
	**Living with person with dementia, n (%)^d^**							
		No	11 (30)		2 (13)		13 (22)	
		Yes	26 (70)		13 (87)		39 (66)	

^a^Not applicable.

^b^Missing data for Brazil: caregiver age, n=7.

^c^Missing data for Brazil: relationship to person with dementia, n=1.

^d^Missing data for Brazil: living with person with dementia, n=7.

#### Attendance

In Brazil, 52% (23/44) of participants attended all 14 sessions, and in India, 53% (8/15) of participants had full attendance.

#### Attrition

In Brazil, the attrition rate was 9% (4/44), denoting the percentage of participants who did not complete the vCST program due to various reasons: caregiver unavailability to support the participant (n=2, 50%), hospitalization due to COVID-19 (n=1, 25%), and to go on a vacation (n=1, 25%). There were no dropouts from vCST groups in India.

#### Outcome Measure Completion

Researchers completed preintervention outcome assessments with all people with dementia; however, some caregivers (3/59, 5%) were unavailable to provide preassessment measures. Retention of people with dementia to follow-up was 89% (39/44) in Brazil and 93% (14/15) in India. In India, 87% (13/15) of caregivers completed all follow-up assessments, but this figure was lower in Brazil (31/44, 70%). This was attributed to the caregivers being occupied with family and work commitments, particularly at a time of increased pressure during the pandemic. In addition, some people with dementia did not have 1 named caregiver and were supported by many family members or paid caregivers who did not always feel that they could provide accurate information. Overall, 68% (40/59) of participant dyads across both sites completed all postintervention outcome measures. No measures caused distress, and no measures had individual items missing.

Pre- and postintervention means and mean differences are outlined in Table 2. Analyses were exploratory and not powered to detect specific changes. The results suggest a small decrease in cognitive ability from baseline to follow-up. Small improvements across quality-of-life domains were observed in people with dementia. We observed moderate reductions in the activity of daily living ability across all domains in both sites. Conflicting outcomes were observed in caregiver burden outcomes, with a small reduction in burden scores on the ZBI but an increase in burden scores according to the Dementia Caregiver Experience Scale measure.

Multimedia Appendix 1 presents results by country. The direction and magnitude of change were similar across both sites; however, notable differences emerged: cognition where the decrease was smaller in India, quality-of-life score (social relationships) where the increase was smaller in India, and quality-of-life score (psychological and environment domains) where a reduction was observed in India compared with an improvement in Brazil.

**Table 2:** Pre- and postintervention outcome measures^a,b^.

Outcome (range)	Preintervention measures			Postintervention measures			Mean improvement (pretest-posttest)			
	Values, n (%)	Values, mean (SD)	Values, n (%)		Values, mean (SD)	Values, n (%)		Mean difference (95% CI)	*P* value	Effect size (95% CI)
ADAS-Cog^c^ (0-70)	59 (100)	27.11 (12.92)	52 (88)		27.36 (14.53)	52 (88)		−1.20 (−3.25 to 0.85)	.24	−0.16 (−0.44 to 0.11)
WHOQOL-BREF^d^: physical health (4-20)	59 (100)	14.48 (2.82)	51 (86)		14.80 (2.56)	51 (86)		0.38 (−0.25 to 1.01)	.23	0.17 (−0.11 to 0.45)
WHOQOL-BREF: psychological (4-20)	59 (100)	14.17 (2.13)	51 (86)		14.75 (2.02)	51 (86)		0.58 (0.04 to 1.11)	.04	0.30 (0.21 to 0.58)
WHOQOL-BREF: social relationships (4-20)	59 (100)	15.12 (1.71)	51 (86)		15.48 (1.96)	51 (86)		0.52 (−0.05 to 1.10)	.07	0.26 (−0.03 to 0.53)
WHOQOL-BREF environment (4-20)	59 (100)	15.03 (1.96)	51		15.25 (1.82)	51 (86)		0.36 (−0.12 to 0.85)	.14	0.21 (−0.07 to 0.49)
ADCS-ADL^e^ (0-78)	44 (100)	44.34 (16.55)	40 (91)		42.00 (16.44)	40 (91)		−3.18 (−5.35 to −1.01)	.005	−0.47 (−0.79 to −0.14)
IADL-EDR^f^—cognitive domain (0-100)	15 (100)	37.65 (19.77)	14 (93)		43.97 (20.72)	14 (93)		−8.64 (−17.91 to 0.64)	.07	−0.54 (−1.09 to 0.03)
IADL-EDR—physical domain (0-100)	15 (100)	4.20 (7.95)	14 (93)		10.57 (15.71)	14 (93)		−7.50 (−16.61 to 1.61)	.10	−0.48 (−1.02 to 0.09)
ZBI^g^ (0-88)	56 (95)	35.02 (18.04)	47 (80)		32.91 (17.69)	46 (78)		1.33 (−0.96 to 3.61)	.25	0.17 (−0.12 to 0.46)
DemCarES^h^ (17-51)	53 (90)	28.94 (6.91)	46 (78)		29.35 (7.28)	43 (73)		−0.67 (−1.90 to 0.55)	.27	−0.17 (−0.47 to 0.13)

^a^Positive maximum scale scores: ADAS-Cog=0, WHOQOL-BREF (Physical health, Psychological, Social relationships, Environment)=20, ADCS-ADL=78, IADL-EDR (Cognitive domain, Physical domain)=0, ZBI=0, DemCarES=17.

^b^Effect size was calculated using Cohen *d* (complete case analysis). No adjustments were made for multiple testing because analyses are exploratory.

^c^ADAS-Cog: Alzheimer’s Disease Assessment Scale-Cognitive Subscale.

^d^WHOQOL-BREF: World Health Organization Quality of Life Brief Version.

^e^ADCS-ADL: Alzheimer’s Disease Cooperative Study-Activities of Daily Living Scale, administered in Brazil only.

^f^IADL-EDR: Instrumental Activities of Daily Living for elderly people, administered in India only.

^g^ZBI: Zarit Burden Interview.

^h^DemCarES: Dementia Caregiver Experience Scale.

### Qualitative Results

#### Overview

A total of 36 qualitative interviews were conducted. In Brazil, 12 people with dementia and their caregivers took part. In India, interviews were conducted with 15 people with dementia and caregivers. This comprises all participants from the first 2 vCST groups in Brazil, and all participants from the 5 groups in India. In addition, 4 group leaders from Brazil took part in interviews, and from India, 3 group leaders and 2 organizational decision makers from the NGO in India. All participants who were invited to the interviews agreed to take part.

Guided by the CFIR, we explored 2 main areas in the analysis: (1) acceptability of vCST and (2) barriers and facilitators to implementation.

#### Acceptability of vCST

All interview participants were asked directly about their experiences of taking part in vCST and were asked to reflect on how it compared to previous face-to-face activities. Overall, participant and caregiver evaluation of vCST was positive, with key benefits relating to providing occupation, enjoyment, and social interaction at the time of isolation:

I liked her activeness and purposefulness...that itself is important. Earlier she used to simply sit but now she has something to do, so that kind of purposefulness is really appreciable.

Caregiver 4, India

We talk and such in the house, but we are only a few here. Now my family is almost just me and him...[the] television doesn’t interact.

Caregiver 8, Brazil

At first I didn’t want to attend the sessions (laughs), I fought, I wanted to hit everyone, but I liked it.

Person with dementia 1, Brazil

The remote delivery and national recruitment in Brazil also enabled the attendance of some participants from outside the urban centers of Rio de Janeiro and São Paulo, where most services are provided:

You’re interacting there from Rio, [name of another participant] there from Itapetininga, the other lady also from another place...with this pandemic business...we don’t need to have physical contact. I think it’s great.

Caregiver 8, Brazil

However, many, in particular, the facilitators who had had the experience of delivering both vCST and face-to-face CST, felt that the social connection and stimulation would have been stronger if the intervention had taken place face to face:

There are many more activities that can be done in person, rather than virtually...like for example, throwing ball to each other, doing physical activities together. Even sensory stimulation like...hearing sounds or seeing things...And I feel just physically being present and seeing other people is definitely...much more helpful.

Group leader 3, India

I think it would have been better if it could have happened in person. But during the COVID situation...this was more helpful and comfortable as anybody can attend from any place. Maybe still, I feel it would have been more beneficial for the dementia group if it were a direct session.

Caregiver 6, India

The participants observed additional issues with intervention acceptability that were related to the participants’ access to suitable technology and computer literacy, which was compounded by cognitive impairment:

The main issue was internet. I would say… so we had only three participants in a group… along with a facilitator and a co-facilitator… which means that, like five different internet connections. So, the problem was if even one participant had a disruption in their internet, it tends to affect the whole group.

Group leader 1, India

At first it was more difficult, because the computer she could use at this time, I was using for work...so she had to do it on her phone...The images were too small for her to see, so that got in the way.

Caregiver 7, Brazil

Group leaders also reflected that it was more difficult to gauge engagement and facilitate a group virtually, as opposed to face to face:

Just knowing the body language, if the person is feeling sleepy, or the person’s not enjoying it and stuff like that. You’re not able to notice it as much because it is a virtual set up.

Group leader 1, India

Sometimes...the participants would talk over other people. We will ask someone a question, and that person...would answer, but then another person would answer also, and the two answers were colliding there, and it was hard to manage that, because it was virtual sessions.

Group leader 2, Brazil

#### Facilitators and Barriers to Implementation

Facilitators and barriers are included in Tables 3 and 4, categorized by CFIR domain with illustrative quotes. Key facilitators included the following:

- Innovation: Facilitators included the evidence base of CST and its advantage over other psychosocial interventions as a manualized intervention, which was also flexible to the needs of the participants. Some group leaders reflected that they were aware of few other interventions for people with dementia taking place virtually at the time.
- Outer setting: An international collaborative effort enabled funding and sharing of protocols and training materials. Many caregivers reflected that they were appreciative that the person with dementia could attend vCST at a time of social isolation due to COVID-19 restrictions.
- Inner setting: Staff in both sites were motivated to offer a service for people with dementia, and many participants reflected on the need for more support for people with dementia. Another facilitator to implementation was the training and supervision of staff at the NGO and trainee psychologists at the university. The NGO in India were able to appoint permanent staff members to take on vCST responsibilities as part of their role and integrate vCST into the existing services and caseload.
- Individuals: Most people with dementia relied on caregivers’ support and would often miss sessions if their caregiver was unavailable. All groups also required 2 group leaders: one to lead the activities and another to provide technological support and to contact caregivers if a participant was struggling to engage. In India, group leaders reflected that adoption of vCST improved if it was suggested to participants by a clinician.
- Processes: Key implementation strategies included providing mock vCST sessions with caregivers and people with dementia to orient them to the platform and posting out activity packs to those who did not have resources at home.

**Table 3:** Facilitators to implementation.

CFIR^a^ domain and subdomains^b^			Quotes
**Innovation**			
	Innovation evidence-base	“In terms of evidence based published literature information… the effectiveness of CST and the cost effectiveness in other centers...That helped in choosing the most appropriate intervention.” [Decision maker 1, India]	
	Innovation relative advantage	“There was this one organization...a day center facility were doing... one-on-one video calls to have some sort of a social interaction during the pandemic.” [Group leader 2, Brazil]	
	Adaptability (of vCST^c^ protocol)	“I think we had flexibility, because as I said one was the education level of patients and then the language that had to be used.” [Decision maker 2, India]	
**Outer setting**			
	Local conditions (need for socializing during lockdowns)	“He was...looking forward to the session, especially social interaction because the pandemic had obviously you know sort of cut down a lot of such interactions.” [Caregiver 1, India]	
	Partnerships and connections (international research partnership)	“We based it ourselves in this protocol, which was already published with some guidelines for developing the CST virtually.” [Group leader 1, Brazil]	
	Financing (international research funding)	“We were able to purchase the items that we need to deliver CST at our center. And for regarding technology...we were able to provide some of the participants with a tablet computer and the data for them.” [Decision maker 1, India]	
**Inner setting**			
	Tension for change (need for psychosocial treatment)	“There is no actual evidence based structured manual intervention available in India prior to this, so this provided as an opportunity to make it available for our patients.” [Decision maker 1, India]	
	Culture—learning centeredness (supporting trainee psychologists, Brazil)	“I really like participating on the project from my experience, in gaining experience, on like clinical experience and also a little bit of research too.” [Group leader 3, Brazil]	
	Compatibility (with service and caseload, India)	“We have a regular clinic so we identify participants from the clinic.” [Decision maker 1, India]	
	Access to knowledge and information (training and supervision)	“We had training, of course, and we also had regular supervision from our supervisor.” [Group leader 1, India]	
	Work infrastructure—staff (at NGO^d^, India)	“Making sure that the facilitators are in substantive posts and not in fleeting positions so they are available for a longer time.” [Decision maker 1, India]	
**Individuals**			
	Opinion leaders (recommendation from doctors)	“If the doctor sometimes says, ‘you should do this, this will be beneficial for you,’ it really helps in the Indian context of the doctor’s word for you.” [Group leader 2, India]	
	Other implementation support—availability or capability (caregivers)	“Some of [the caregivers] would stay next to the person living with dementia...especially when the person was a little bit shy, [or] had more difficulty with technology...They were...mediating this communication.” [Group leader 2, Brazil]	
	Other implementation support—availability or capability (cofacilitator)	“One of the psychologists is delivering the session, and we need someone to support us at the technical end, we need someone to support us.” [Group leader 2, India]	
	Intervention recipient—need (person with dementia—need to stay home and subsequent isolation)	“Some of these people would not have come for in-person CST, because they could not afford transportation, did not have proper transportation, were frail, or had some kind of physical comorbidity or pain.” [Group leader 2, India]	
**Implementation process**			
	Tailoring strategies (mock vCST sessions and activity packs)	“We do have one trial session, where I sit with them individually. And then we have one group trial session, to see if they’re comfortable in a group.” [Group leader 2, India] “We posted the materials...for number games, we had paper sheets. And colouring papers and some origami papers...We took printouts and posted it to their house.” [Group leader 1, India]	

^a^CFIR: Consolidated Framework for Implementation Research.

^b^Context-specific descriptions are given in parentheses.

^c^vCST: virtual cognitive stimulation therapy.

^d^NGO: nongovernmental organization.

**Table 4:** Barriers to implementation.

CFIR^a^ domain and subdomains^b^			Quotes
**Innovation**			
	Adaptability (virtual delivery of CST^c^)	“I was running face-to-face sessions before they started [vCST]. Face-to-face CST was great...my group ran with eight members, six to eight, consistently. So, I had a huge group coming every Friday. It was amazing, they could form more connections, and turn taking is a little bit easier...It’s a little harder like you with the Zoom.” [Group leader 2, India	
	Innovation design (need for marketing)	“It doesn’t have much publicity. If it wasn’t for chance, if this person hadn’t put us in, I wouldn’t have made it. So, I think in terms of dissemination it could be broader.” [Caregiver 4, Brazil]	
**Outer setting**			
	Critical incidents (COVID-19 pandemic)	“It was COVID and people are falling sick...even the facilitators are sick, at some point.” [Group leader 1, India]	
	Local attitudes (dementia awareness)	“In Brazil, I think it’s a cultural thing to think that dementia symptoms it’s part of a natural aging...So, when older people, and people living with dementia...come to a doctor to be evaluated they sometimes don’t have mild symptoms anymore.” [Group leader 2, Brazil]	
	Local attitudes (traditional focus on medical model)	“People weren’t aware of psychosocial interventions for dementia prior to this. They had very different model for working with people with dementia.” [Decision maker 1, India]	
	Local conditions (access to technology)	“Most of the people that we had in the groups were from the south eastern region. And that’s kind of a more developed region financially...I think today most people in Brazil have access to internet. Maybe not their computer, but maybe cell phones and something like that.” [Group leader 3, Brazil]	
**Inner setting**			
	Structural characteristics—work infrastructure (staff availability)	“When we think of scaling it up, we might have to do it first of all in institutions where there is enough manpower of mental health professionals to deliver the CST...dementia care in India is still mental health care and we’re still very under-resourced as far as manpower is concerned.” [Decision maker 2, India]	
**Individuals**			
	High-level leaders—capability (lack of dementia awareness)	“Some of the policymakers, who we interviewed at the beginning [in previous stakeholder engagement] weren’t even aware of the issues relating to dementia.” [Decision maker 1, India]	
	Intervention deliverers—capability (basics in clinical skills needed)	“I think we if we didn’t have the training, it would be very hard to just come to the groups...I didn’t have any contact [with people with dementia] before.” [Group leader 3, Brazil]	
	Intervention recipients—capability (sensory impairment and computer literacy)	“So, one challenge was delivering it virtually. My mother was not able to hear very well. Now she has a hearing aid, she has the headphones but still that was a part of a problem of communication.” [Caregiver 5, India] “I don’t know how to use the computer (laughs).” [Person with dementia 10, Brazil]	
**Implementation process**			
	Assessing needs—innovation recipients (severity of dementia)	“If you have some difference in severity of dementia, because the activities demand something, and maybe it can be boring for who is not so severe.” [Group leader 4, Brazil]	
	Assessing needs—innovation recipients (baseline assessments)	“The first is, I think, the baseline evaluations were very long, and that was kind of hard on the, not on the people with dementia, but on their family members, the caregivers.” [Group leader 3, Brazil]	
	Reflecting and evaluating—implementation (lack of long-term follow-up)	“One question that most people with dementia their caregivers made was, if it was possible to have more than 14 sessions. So maybe adapting the maintenance CST for the virtual program. I think it would be a suggestion for the future.” [Group leader 1, Brazil]	

^a^CFIR: Consolidated Framework for Implementation Research.

^b^Context-specific descriptions are given in parentheses.

^c^CST: cognitive stimulation therapy.

Key barriers related to the 5 CFIR domains are as follows:

- Innovation. Most group leaders highlighted challenges with the online delivery of CST in terms of facilitating a group effectively, meeting individual needs, and supporting participants with the videoconferencing platform. Many leaders reflected on the comparative ease of facilitating a group in person. These issues are outlined in detail in the
  Acceptability of vCST
  section. Finally, group leaders and caregivers highlighted the need for marketing to raise awareness of vCST
- Outer setting. While COVID-19 necessitated and possibly facilitated the online delivery of CST, staff and participant illness during the pandemic was a barrier to group delivery and attendance. Staff at both sites reflected on a lack of dementia awareness, resulting in participants presenting later to clinical services, which is a barrier to recruiting participants with mild to moderate dementia. Similarly, group leaders and decision makers reflected on a lack of awareness of psychosocial interventions for dementia, with the medical model tending to prevail. Finally, in both sites, it was highlighted that poor or limited access to technology is a barrier to involvement.
- Inner setting. The limited availability of mental health personnel was highlighted as a barrier to the wider scale-up of vCST in India.
- Individuals. People with dementia and caregivers faced barriers to taking part in vCST, including a lack of computer literacy and sensory impairment that impacted engagement. Organizational decision makers in India reflected on the lack of dementia awareness within high-level policy makers.
- Processes. One group leader reflected on the length and burden of the baseline assessments on people with dementia and caregivers. Many people with dementia and caregivers expressed a wish for the vCST groups to continue beyond the 14 sessions. At the NGO in India, it was possible to follow up with patients on the caseload; however, group leaders in Brazil wished to be able to continue to support participants and caregivers.

## Discussion

### Principal Findings

We found that it was feasible and acceptable to deliver CST virtually in Brazil and India. We recruited 91% (59/65) of the target sample and were able to run 17 vCST groups. Attrition was low (4/49, 7%), and attendance was moderate, with 53% (31/59) of participants attending all 14 sessions. This is in contrast to a previous trial of face-to-face CST in Brazil, where attrition was similar (6%) but attendance was high (mean 12.8 sessions, median 14 sessions) [39]. In a previous pilot study of face-to-face CST in Chennai, India, attrition was higher with 3 out of 9 participants dropping out [16]. However, these comparisons should be interpreted with care due to small sample sizes and the impact of COVID-19 in both countries.

Outcome measure completion was slightly lower than the target of 75%, as only 68% (40/59) of participant dyads completed all follow-up outcome measures, suggesting a possible measurement burden. Small improvements across all quality-of-life domains were observed in people with dementia. All results should be interpreted with care, as the study was not controlled. Any changes cannot be ascribed to the vCST intervention specifically, and the impact of COVID-19 and consequent social isolation may have played a role in pre- and postintervention measurement changes.

There were some differences in the barriers and facilitators to implementation across the 2 sites. vCST was delivered in an NGO in Chennai, where participants could be recruited from the patient caseload. In Brazil, vCST was delivered through a university where recruitment took place from the community and memory clinics and NGOs who were partnered with the study. Although staff turnover was a barrier to implementation in the NGO in India, decision makers reflected that it would be possible to build CST or vCST into services due to its compatibility with current ways of working. This may have been more of a challenge in the university setting in Brazil, where there is no patient caseload or clinic infrastructure. However, there is scope to build partnerships with community organizations and clinics to recruit participants. Upskilling trainee psychologists to deliver vCST in Brazil also presents a low-cost and scalable solution to implementing vCST in a university setting. Similar solutions have been used for other psychosocial interventions for dementia in other countries [40,41].

A major barrier in both sites was poor or limited access to technology and computer literacy. This issue was also highlighted in studies of vCST conducted in the United Kingdom [23,24]. A survey of the digital divide in India found that just 38% of households are digitally literate [42]. Access is higher in Brazil, where 80% of households have internet access [43]. In both countries, digital access intersects with age, gender, education level, and ethnicity [42-44]. While virtual interventions provide service access to those living in geographically isolated locations, the digital divide is greater in rural areas; in Brazil, only 53% of the rural population have internet access, while 88% in urban areas have internet access [43], and the figures are lower overall in India where in rural areas, only 31% of the population use the internet, while in the urban areas, the percentage rises to 67% [42]. To overcome the barriers to technology access in India, the NGO loaned tablets to participants, which required sufficient funding and resources. We addressed the issues related to digital literacy by implementing the following measures: (1) group leaders provided mock vCST sessions to familiarize participants with the videoconferencing platform, (2) a coleader was available specifically for technology support, and (3) groups sizes were smaller so that all participants could be viewed on the screen at once (average 3.6 participants compared with 6-8 according to the original CST protocol [12]).

Most people with dementia were reliant on caregivers’ technical support to use the videoconferencing technology, and in some cases, caregivers remained present throughout the group sessions. This raises a key issue for those without caregivers, who could be systematically excluded from taking part in virtual psychosocial interventions. If vCST were implemented as a dyadic intervention, this could improve caregivers’ awareness of dementia and person-centered approaches, which is important given the limited number of dementia awareness programs in LMIC settings [45]. However, it could also negatively impact the engagement of the person with dementia, as one of the proposed mechanisms of action of CST is the supportive learning environment, where people with dementia support each other without judgment or embarrassment [46]. If vCST is delivered dyadically, we recommend that participants are briefed at the start of the program to set expectations about the caregivers’ level of involvement in the vCST sessions and that people with dementia are provided with opportunities to take part in activities and discussions alone. Further research could explore the impact of dyadic delivery on outcomes for people with dementia and their caregivers.

### Limitations

In both sites, it is likely that the sample was not representative of the broader population of people living with dementia and their caregivers. Specifically, in Brazil, the sample comprised mostly White individuals (35/44, 80%), which does not reflect the majority Black and mixed Brazilian population. Most participants were from the urban region of southeast Brazil, although the remote method of recruitment did enable participation from areas outside of this region, which were underserved in terms of research and clinical services. In India, all participants were recruited from the same region and were already attending clinical services; this might have resulted in a sample skewed to those with the means to access services.

Online delivery may result in a self-selecting sample, who are more likely to be educated to a higher level and more affluent than the broader population. The mean number of years of schooling of our sample was 11.5 (SD 1.2) in Brazil and 13.0 (SD 2.6) in India. This compares to a national average of 2.5 in Brazil and 1.4 in India for the population aged ≥25 years in 1970 and 1971, respectively [47] although there is huge regional variation in education levels in both countries. To overcome issues related to digital exclusion in India, tablets were loaned to those who needed them. However, in Brazil, people without access to their own technology were excluded.

In terms of the qualitative component, most themes and quotes from a participant perspective were collected from caregivers rather than people with dementia. This is because cognitive impairment affected their recall of sessions. Despite this, caregivers and group leaders reflected on the perceived participant experience of vCST sessions. Interviews took place with all caregiver dyads from India, but only those from the first 2 vCST groups in Brazil due to staff availability. However, the reflections from group leaders and organizational decision makers relate to all vCST groups. Interviews with group leaders and decision makers were carried out in English by a UK-based researcher who was not a member of the immediate research team. This was to limit response bias and encourage honest and critical feedback; however, it limited the interview to people who speak English and may have compromised the representation of non-English speakers.

Finally, the vCST intervention was tested in 2 sites, a university in Brazil and an NGO in India, resulting in lessons for implementation that could be explored in other sites and countries; nevertheless, we acknowledge the limited generalizability of these findings.

### Future Research

To date, vCST has only been trialed within a pandemic context. While this was acceptable to participants during a time of social isolation, many caregiver dyads and group leaders expressed a desire for CST to take place in person. Future research could explore the feasibility of vCST outside of the pandemic context, perhaps specifically targeting those who cannot access in-person services due to limited mobility, health issues, or geographic isolation.

In addition, although there is a strong evidence base for in-person CST, we do not know if the benefits to cognition and quality of life are conferred to the same level over online delivery. A recent feasibility study of vCST (in press Spector, 2023) has shown that a full-scale randomized controlled trial is warranted

### Conclusions

The 14-session vCST program for people with dementia was successfully trialed in a university setting in Brazil and in an NGO in Chennai, India. vCST offered a feasible alternative to in-person groups during the period of pandemic restrictions with potential benefits to quality of life, but there were barriers related to technology access and computer literacy. Outside of the pandemic context, vCST could be provided to people with dementia who are geographically isolated or who have mobility- or health-related difficulties.

## Appendix

Multimedia Appendix 1 Pre- and postintervention outcome measures by country.

## Abbreviations

CFIR: Consolidated Framework for Implementation Research
CST: cognitive stimulation therapy
LMIC: low- and middle-income countries
NGO: nongovernmental organization
vCST: virtual cognitive stimulation therapy
ZBI: Zarit Burden Interview
