# Supplementary material for: Online Cognitive Stimulation Therapy for Dementia in Brazil and India: Acceptability, Feasibility, and Lessons for Implementation
Source: JMIR Aging. 2024 Jun 11;7:e55557. doi: 10.2196/55557 (PMC11200045; doi:10.2196/55557)
Supplement: Multimedia Appendix 1 [file aging_v7i1e55557_app1.docx]

# Supplementary file 1: Pre- and post-intervention outcome measures by country

| **Outcome (range)** | **India** | | | | | | | | **Brazil** | | | | | | | |
| --- | --- | --- | --- | --- | --- | --- | --- | --- | --- | --- | --- | --- | --- | --- | --- | --- |
|  | **Pre-intervention** | | **Post intervention** | | **Mean improvement (pre-post)** | | | | **Pre-intervention** | | **Post intervention** | | **Mean improvement (pre-post)** | | | |
|  | **n** | **Mean (SD)** | **n** | **Mean (SD)** | **n** | **Mean difference (95% CI)** | **p-value** | **Effect size (95% CI)** | **n** | **Mean (SD)** | **n** | **Mean (SD)** | **n** | **Mean difference (95% CI)** | **p-value** | **Effect size (95% CI)** |
| ADAS-Cog (0-70) | 15 | 19.50 (7.43) | 14 | 19.43 (9.97) | 14 | -0.52  (-5.51, 4.47) | 0.83 | -0.06  (-0.58, 0.47) | 44 | 29.70  (13.42) | 39 | 29.89  (14.96) | 39 | -1.42  (-3.64, 0.80) | 0.20 | -0.21  (-0.52, 0.11) |
| WHOQOL-BREF: Physical health (4-20) | 15 | 14.32 (2.23) | 14 | 14.94  (2.27) | 14 | +0.37  (-0.65, 1.38) | 0.45 | +0.21  (-0.33, 0.74) | 44 | 14.53  (3.01) | 37 | 14.75  (2.68) | 37 | +0.39  (-0.42, 1.19) | 0.34 | +0.16  (-0.17, 0.48) |
| WHOQOL-BREF: Psychological (4-20) | 15 | 13.87  (1.68) | 14 | 13.81  (2.13) | 14 | -0.05  (-0.93, 0.84) | 0.91 | -0.03  (-0.56, 0.49) | 44 | 14.27  (2.27) | 37 | 15.10  (1.89) | 37 | +0.81  (0.15, 1.47) | 0.02 | +0.41  (0.07, 0.74) |
| WHOQOL-BREF: Social relationships (4-20) | 15 | 14.93  (1.61) | 14 | 14.95  (1.40) | 14 | +0.10  (-0.78, 0.97) | 0.82 | +0.06  (-0.46, 0.59) | 44 | 15.18  (1.75) | 37 | 15.68  (2.12) | 37 | +0.68  (-0.05, 1.42) | 0.07 | +0.31  (-0.02, 0.64) |
| WHOQOL-BREF Environment (4-20) | 15 | 15.00  (2.01) | 14 | 14.82  (1.67) | 14 | -0.18  (-1.27, 0.91) | 0.73 | -0.10  (-0.62, 0.43) | 44 | 15.03  (1.97) | 37 | 15.42  (1.87) | 37 | +0.57  (0.03, 1.11) | 0.04 | +0.35  (0.02, 0.68) |
| IADL – Cognitive domain (0-100) | 15 | 37.65 (19.77) | 14 | 43.97 (20.72) | 14 | -8.64  (-17.91, 0.64) | 0.07 | -0.54  (-1.09, 0.03) |  | | | | | | | |
| IADL – Physical domain (0-100) | 15 | 4.20 (7.95) | 14 | 10.57 (15.71) | 14 | -7.50  (-16.61, 1.61) | 0.10 | -0.48  (-1.02, 0.09) |  |  |  |  |  |  |  |  |
| ADCS-ADL (0-78) |  | | | | | | | | 44 | 44.34 (16.55) | 40 | 42.00 (16.44) | 40 | -3.18  (-5.35, -1.01) | 0.005 | -0.47  (-0.79, -0.14) |
| ZBI (0-88) | 15 | 26.67  (12.83) | 14 | 25.36  (13.56) | 14 | +1.64  (-2.79, 6.08) | 0.44 | +0.21  (-0.32, 0.74) | 41 | 37.71  (19.03) | 33 | 36.12  (18.43) | 32 | +1.18  (-1.63, 4.00) | 0.40 | +0.15  (-0.20, 0.50) |
| DemCarES (17-51) | 12 | 26.83  (3.16) | 14 | 26.93  (3.17) | 12 | -0.25  (-2.84, 2.24) | 0.84 | -0.06  (-0.63, 0.51) | 41 | 29.56  (7.59) | 32 | 30.41  (8.30) | 31 | -0.84  (-2.30, 0.63) | 0.25 | -0.21  (-0.56, 0.15) |
| **Abbreviations:** ADAS-Cog: Alzheimer's Disease Assessment Scale–Cognitive Subscale; WHOQOL-BREF: World Health Organization Quality of Life Brief Version; ADCS-ADL: Alzheimer’s Disease Cooperative Study–Activities of Daily Living Scale; IADL-EDR: Scale for the Instrumental Activities of Daily Living in the Elderly; ZBI: Zarit Burden Interview; DemCarES : Dementia Caregiver Experience Scale.  ADCS-ADL administered in Brazil only, and IADL-EDR in India. The positive maximum scale score is underlined.  Effect size calculated using Cohen’s d (complete case analysis). No adjustments were made for multiple testing because analyses are exploratory. | | | | | | | | | | | | | | | | |
